# The RNA-Binding Protein RBMX Mediates the Immunosuppressive Microenvironment of Osteosarcoma by Regulating CD8^+^T Cells

**DOI:** 10.3390/cancers17172928

**Published:** 2025-09-06

**Authors:** Yu Qiu, Chao Pu, Chengguang Wang, Zhengxue Quan

**Affiliations:** 1 Department of Orthopaedic Surgery, The First Affiliated Hospital of Chongqing Medical University, Chongqing 400016, China; qiuyu840205@163.com (Y.Q.); 19923706512@163.com (C.W.); 2 Chongqing Municipal Health Commission Key Laboratory of Musculoskeletal Regeneration and Translational Medicine, Chongqing 400016, China; 3 Beijing Anzhen Nanchong Hospital of Capital Medical University & Nanchong Central Hospital, Nanchong 637000, China; puchao3310@sina.com

**Keywords:** osteosarcoma, RBMX, TME, CD8^+^T cells, prognosis

## Abstract

**Background:** The progression of osteosarcoma is closely related to the immune microenvironment. Related studies have found that the RNA-binding motif protein, X-linked (RBMX), plays a regulatory role in modulating the biological characteristics of the tumor microenvironment (TME). However, its regulatory mechanism in osteosarcoma remains unclear. **Methods:** In this study, the expression of RBMX in osteosarcoma was analyzed using the results of bulk and single-cell transcriptome sequencing of human osteosarcoma. The RBMX knockout cell line was constructed via lentivirus transfection. The mouse subcutaneous implantable tumor model and single-cell transcriptome sequencing analysis revealed the effects of RBMX on the osteosarcoma microenvironment, as verified via multiplex immunofluorescence, flow cytometry, and PCR experiments. **Results:** Using the TARGET database and multiplex immunofluorescence, we found that RBMX is highly expressed in human osteosarcoma and is associated with poor prognosis. The high expression of RBMX may mediate the immunosuppressive microenvironment of human osteosarcoma. In vitro cell experiments showed that knockout of RBMX significantly inhibited the proliferation of mouse osteosarcoma cells. Through single-cell transcriptome sequencing analysis of subcutaneous implantable tumors in mice, we determined that RBMX deletion substantially elevated the recruitment of cytotoxic CD8^+^T cells within the mouse TME, which was further verified through flow cytometry analysis. Cell coculture assay confirmed that knockout of RBMX significantly enhanced the cytotoxic activity of CD8^+^T cells. Finally, cell communication and in vitro experimental verification revealed that knocking out RBMX might enhance the infiltration of CD8^+^T cells by upregulating histocompatibility 2, K1, and K region (H2-K1) and downregulating thrombospondin 1 (THBS1). **Conclusions:** This study may provide potential targets for reshaping the immune microenvironment of osteosarcoma and improving its therapeutic efficacy.

## Simple Summary

This study found that RBMX is highly expressed in human osteosarcoma. Through animal models combined with single-cell transcriptome sequencing analysis, it was discovered that knockout of RBMX significantly enhanced the infiltration of immune cells in the tumor microenvironment, suggesting that intervening in the expression of RBMX may help make tumors more likely to respond to immunotherapy. This study reveals that CD8^+^T cells may be important cells regulated by RBMX. Further experiments revealed that RBMX regulates the expression of H2-K1 and THBS1, driving the depletion of CD8^+^T cells in osteosarcoma through cell communication. This study may provide potential targets for reshaping the immune microenvironment of osteosarcoma and improving the therapeutic effect of osteosarcoma.

## 1. Introduction

Osteosarcoma is an aggressive primary bone malignancy [1]. The proportion is highest among those aged 10 to 24, with an incidence rate of approximately 0.0003% to 0.0005% among men and about 0.0002% to 0.0004% among women [2]. The average 5-year survival rate of patients with local osteosarcoma is approximately 80%; however, for those with metastatic disease, it is as low as 20% to 30% [2,3]. Although innovations in comprehensive treatments such as immunotherapy, targeted therapy, chemotherapy, and surgery have prolonged the survival time, the prognosis remains unsatisfactory [4]. Therefore, exploring new therapeutic targets and enhancing therapeutic efficacy to improve the prognosis of patients are important tasks in current research.

Evidence indicates that the TME significantly influences the invasiveness and treatment response of osteosarcoma [5]. Osteosarcoma progression is fueled by the continuous interplay between tumor cells and their microenvironment, resulting in suppressed antitumor immunity and increased metastatic potential [6]. Studies have shown that TME remodeling is associated with poor prognosis and chemotherapy resistance [7]. For example, the high infiltration of M2 macrophages is associated with a poor survival rate [8], while extracellular matrix components derived from fibroblasts promote invasion [9]. In addition, relevant studies have found that mifamurtide can improve the survival rate of osteosarcoma patients by regulating the TME status [10,11]. Research has found that a PD-1 monoclonal antibody Pembrolizumab can effectively activate CD8^+^T cells in the osteosarcoma microenvironment and inhibit the progression of osteosarcoma [12]. Similarly, a clinical trial found that targeted TAM therapy can effectively alleviate the progression of osteosarcoma [13]. These findings emphasize the potential of the TME as a therapeutic target. Therefore, further exploration of the new mechanism of TME regulation in osteosarcoma may help to improve the prognosis of patients with osteosarcoma.

Recent research underscores the crucial regulatory function of N6-Methyladenosine modification in the TME [14], with increasing focus on the involvement of RNA-binding proteins (RBPs) in TME regulation [15,16]. RBP regulates the infiltration of tumor microenvironment cells by influencing the splicing, stability, and translation of target RNA, thereby regulating the state of the tumor microenvironment. Studies have shown that RBPs reshape the immunosuppressive microenvironment by regulating tumor-associated macrophage polarization, T cell infiltration, and extracellular matrix remodeling [17,18,19]. The RNA-binding motif protein X (RBMX), as an important RBP, has been reported to have a pro-cancer effect in various cancers [20]. RBMX can affect the invasion, metastasis, and regulation of the immune microenvironment of tumor cells. For example, RBMX-mediated adipogenesis promotes the metastasis of liver cancer [21]. RBMX binds to miR-19b-3p and promotes distant metastasis of lung cancer [22] and it also drives the M2 polarization of tumor-associated macrophages (TAMs), thereby accelerating colorectal cancer progression [23]. However, there are currently few relevant research reports on the exploration of RBMX in osteosarcoma. Thus, the potential involvement of RBMX in osteosarcoma progression through TME modulation requires further investigation, particularly regarding its underlying molecular mechanisms. Elucidating RBMX-mediated immunoregulatory pathways could provide critical insights into osteosarcoma immune evasion and inform novel immunotherapeutic approaches.

In this study, the expression of *RBMX* in osteosarcoma was identified using the TARGET database and clinical samples. Subcutaneous tumor models were constructed by *RBMX* gene knockout mouse osteosarcoma cell lines, and the key cell groups regulated by them were identified by single-cell transcriptome sequencing analysis. Finally, the mechanism by which RBMX regulates the immune microenvironment state was explored through cell communication analysis and PCR experiments, providing a scientific basis for the treatment and prognosis of osteosarcoma.

## 2. Materials and Methods

### 2.1. Single-Cell Transcriptome Sequencing and Bulk Sequencing Analysis of Human Osteosarcoma

Human osteosarcoma single-cell RNA sequencing data were sourced from the GEO repository (GSE162454, *n* = 6 cases). The dataset was downloaded from the GEO database, and the single-cell matrix was constructed using the Seurat package (v4). The criteria for screening low-quality cells were as follows: genes detected in a single cell > 200 and percentage of mitochondrial genes < 10%. We normalized the data with log-normalization before use to correct the batch processing effect and sequencing depth. PCA was conducted on the top 2000 highly variable genes, and harmony was used to eliminate the batching effect. Cell types were identified based on specific markers: myeloid cells (LYZ, S100A9), T/NK cells (CD3D, CD3E, NKG7), fibroblasts (ACTA2, TAGLN), osteoblasts (ALPL, RUNX2, CLEC11A), B cells (CD79A, MS4A1), and endothelial cells (VWF, PECAM1) [24,25]. Bulk transcriptomic profiles were acquired from the TARGET database, with subsequent normalization and analysis performed using TPM quantification. For the TARGET cohort, a total of 95 primary treated high-grade osteosarcoma tumor samples without metastasis were included. For prognosis analysis, patients with a prognosis of less than 30 days were excluded. Among them, 18 cases did not relapse, 32 cases relapsed or metastasized, and the rest lacked information in this regard.

### 2.2. Single-Cell Transcriptome Analysis of Mouse Samples

The single-cell transcriptome sequencing of mice was commissioned from LC-Bio Technologies (Hangzhou, China) Co., Ltd. One sample of subcutaneous tumors in LM8 and LM8-KO cells, respectively, was taken for single-cell transcriptome sequencing analysis. CD45+ cells and CD45− cells were isolated using fluorescence-activated cell sorting (FACS). The positive gate for CD45 was rigorously set based on unstained control cells, ensuring that the false-positive rate was maintained below 1%, and the above cells were mixed at a ratio of 8:2 for single-cell transcriptome sequencing. This single-cell RNA sequencing experiment based on the chemical version of 10× Genomics Chromium v3.1 was successful. The high sensitivity characteristic of this chemical version ensured the output of high-quality data. After strict quality control, the wild group obtained 328,331,236 Raw Reads from the original data before quality control and the proportion of high-quality and valid data obtained after quality control was 83.7%. A total of 10,376 high-quality cells were retained. The knockout group obtained 346,703,569 Raw Reads from the raw data before quality control, and the proportion of high-quality valid data obtained after quality control was 90.36%. A total of 11,208 high-quality cells were retained. The criteria for screening low-quality cells were as follows: genes detected in a single cell > 200 and percentage of mitochondrial genes < 20%. We normalized the data with log-normalization before use to correct the batch processing effect and sequencing depth. PCA was conducted on the top 2000 highly variable genes, and harmony was used to eliminate the batching effect. Cell types were identified based on specific markers: macrophages (CD14, CD68), neutrophils (CD14, CXCR2), B cells (CD45, CD79A, MS4A1), NK cells (CD45, NCR1), CD4^+^T cells (CD45, CD3D, CD3E, CD4), and CD8^+^T cells (CD45, CD3D, CD3E, CD8) [26]. Differentially expressed genes (DEGs) between clusters were determined using Findmarkers, with criteria of an adjusted *p*-value < 0.05 and |log2FC| > 0.25. Cell communication analysis was conducted using CellChat [27]. Functional enrichment analysis of DEGs was performed using [28] (v4.4.4) with the KEGG and GO databases, applying an FDR threshold of <0.05.

### 2.3. Transcriptome Data Analysis of Mouse Osteosarcoma Cells

Bulk transcriptome sequencing was performed on the mouse cell lines LM8, LM8-KO, K7M2, and K7M2-KO, and three samples in each group were repeatedly tested. Total RNA was extracted from the collected mouse osteosarcoma cells using TRIzol [19]. Transcriptome sequencing analysis was performed by LC-Bio Technologies (Hangzhou, China) Co., Ltd. using the Illumina NovaSeq 6000 platform. Bulk RNA-seq reads were aligned to the mouse reference genome (GRCm39) using HISAT2 (version 2.2.1). Gene expression quantification was performed against the GENCODE (version M32) comprehensive gene annotation. Transcripts per million (TPM) values were used for downstream analysis.

### 2.4. Multiplex Immunofluorescence Staining of Human Osteosarcoma Tissues and Subcutaneous Tumor Tissues of Mouse Osteosarcoma

Tumor tissue samples were collected from 79 osteosarcoma patients at the First Affiliated Hospital of Chongqing Medical University. The samples were fixed in formalin and subsequently embedded in paraffin blocks. Subcutaneous tumor tissues from mice were similarly dissected, fixed in formalin, and embedded in paraffin blocks. Multiplex immunofluorescence (mIF) was performed on paraffin-embedded tissue. After deparaffinization using xylene and graded ethanol, heat-induced epitope retrieval was conducted in Tris-EDTA buffer (pH 9.0) at 95 °C for 20 min. Endogenous peroxidase activity was inhibited using 3% hydrogen peroxide for 10 min, followed by a 30 min blocking of nonspecific binding with a serum-free protein block solution. For the staining procedure, we added the primary antibody and incubated it overnight at 4 °C in a humidified chamber. After rinsing, the HRP-labeled secondary antibody was applied, followed by a 1 h incubation at room temperature and a subsequent wash. For the second primary antibody staining, we applied a TSA fluorescent dye solution, incubated it in the dark at room temperature for 10 min, followed by thermal repair using a 10 mm sodium citrate buffer at 95 °C for 10 min, and washed the slides. Finally, DAPI staining and sealing were carried out and observed under a microscope. All reagents (including primary and secondary antibodies) were provided by Wuhan Servicebio Technology Co., Ltd. (Wuhan, China). The primary antibodies included CD8A, RBMX, and PanCK. This study evaluated RBMX protein expression in osteosarcoma specimens using a standardized immunohistochemical scoring system. The staining intensity was graded as 0 (negative), 1 (weak), 2 (moderate), or 3 (strong) in this study, while the percentage of positive tumor cells was scored from 0 (<5%) to 4 (>75%) based on our criteria. In the present study, the final immunoreactivity score (range 0–12) was calculated by multiplying the intensity score by the percentage score. This study classified samples as RBMX-low (score ≤ 4) or RBMX-high (score > 4) based on this composite assessment. The infiltration level of immune cells is determined by the proportion of positive immune cells to the total number of cells in the field of view. To ensure objectivity, this research employed two blinded pathologists who independently scored at least five representative high-power fields per case, with discrepancies resolved through consensus review. The current study utilized this semi-quantitative approach to achieve the objective and reproducible evaluation of RBMX expression patterns in a human osteosarcoma cohort.

### 2.5. Cell Culture

Mouse osteosarcoma cell lines (K7M2, LM8) were purchased from Haixing Biosciences (Suzhou, China). Both K7M2 and LM8 were maintained in high-glucose DMEM (GIBCO, Grand Island, NY, USA) with 10% fetal bovine serum, at 37 °C, in a 5% CO_2_ humidified incubator. All cell lines used in this study were authenticated by short tandem repeat (STR) profiling and confirmed to be mycoplasma-free. Mycoplasma contamination was routinely monitored every month using a PCR-based detection kit (MEC, HY-K0553). All tests returned negative results throughout the course of the research.

### 2.6. RBMX Knockout Cells

Lentivirus particles for knocking out the RBMX gene via CRISPR/Cas9 were purchased from Genechem Co., Ltd. (Shanghai, China). The construction of the cell lines was carried out in accordance with the instructions and passed the puromycin positive screening (Beyotime, Shanghai, China).

### 2.7. Western Blot

Cells were lysed in RIPA buffer with protease inhibitors, and protein concentrations were measured using the BCA method. Equal amounts of protein (10 μg per lane) were separated using 10% SDS-PAGE and transferred to PVDF membranes. Membranes were then blocked with 5% non-fat milk and incubated overnight at 4 °C with primary antibodies targeting RBMX (1:1000, Abcam, ab190352, Waltham, MA, USA) and β-actin (1:2000, Abcam, ab8226), followed by HRP-conjugated secondary antibodies. Protein bands were visualized using enhanced chemiluminescence and quantified via densitometry using ImageJ software (v1.47). Western blot analysis was performed with three independent biological replicates, with each replicate representing cells harvested from a separate culture grown and treated independently.

### 2.8. Real-Time Quantitative PCR

Total RNA was extracted with TRIzol reagent (NCM Biotech, Suzhou, China), with subsequent cDNA generation using PrimeScript RT reagents (Thermo Fisher, Waltham, MA, USA). Quantitative amplification was performed in triplicate using SYBR Select Master Mix on a Bio-Rad CFX96 platform. The thermal profile included an initial denaturation at 95 °C for 30 s, followed by 40 cycles of 95 °C for 5 s and 60 °C for 30 s. Expression normalization was performed against β-actin using the comparative Ct method. β-actin: Forward Primer GTGACGTTGACATCCGTAAAGA, Reverse Primer GCCGGACTCATCGTACTCC. H2-K1: Forward Primer ACCAGGACTACG CCTACGA, Reverse Primer AACCAGGACGACGTCG. THBS1: Forward Primer CCTGCCAGGGAAGCAACAA, Reverse Primer ACAGTCTATGTAGAGTTGAGCCC. Real-time quantitative PCR analysis was performed with three independent biological replicates, with each replicate representing cells harvested from a separate culture grown and treated independently. Student’s *t*-test was used for analysis. A *p* value < 0.05 was considered statistically significant.

### 2.9. 5-Ethynyl-2′-Deoxyuridine (EdU) Cell Proliferation Assay

EdU binds to DNA strands during the DNA synthesis phase (S phase) of cells through its alkyne groups; then, it covalently binds to fluorescently labeled azides through click chemical reactions, achieving fluorescent labeling and quantitative detection of proliferating cells. Cellular proliferation was assessed using the EdU incorporation assay (Beyotime Biotechnology, Shanghai, China). Cells were incubated for 2 h with 10 μM EdU labeling solution, fixed using 4% paraformaldehyde, and permeabilized with 0.5% Triton X-100. For detection, we utilized Alexa Fluor 594-conjugated click chemistry reagents, while Hoechst 33,342 was employed for nuclear counterstaining. Quantitative analysis was conducted via flow cytometric evaluation. EdU cell proliferation assay was performed with three independent biological replicates, with each replicate representing cells harvested from a separate culture grown and treated independently. Student’s *t*-test was used for analysis. A *p* value < 0.05 was considered statistically significant.

### 2.10. Mouse Subcutaneous Tumor Model

Subcutaneous tumor models of the K7M2 cell line were developed using female BALB/c mice. Female C3H mice were used to construct subcutaneous tumor models of the LM8 cell line. The mice were purchased from Beijing Vital River Laboratory Animal Technology Co., Ltd. (Beijing, China). All mice were housed under specific pathogen-free (SPF) conditions throughout the study. In this study, mice were randomly assigned to experimental groups using a computer-generated randomization sequence to ensure unbiased group allocation. Prior to randomization, all mice were matched for age (6 weeks old), sex, weight (18 g ± 10%), and strain (C3H/C57BL/6) to minimize baseline variability. The randomization sequence was generated using GraphPad Prism (v10), with each mouse assigned a unique identifier to prevent selection bias. To enhance rigor, researchers performing experiments and data collection were blinded to group assignments until after data analysis. Group sizes (*n* = 4/group) were determined based on power calculations from preliminary data or the previous literature. Randomization was verified by confirming no statistically significant differences (*p* > 0.05) in baseline characteristics between groups. This standardized randomization approach ensured unbiased group allocation and methodological reproducibility in accordance with ARRIVE 2.0 guidelines. BALB/c mice (6 weeks old) were subcutaneously injected with 1 × 10^7^ RBMX-knockout or control cells suspended in 50% Matrigel. C3H mice (6 weeks old) were subcutaneously injected with 5 × 10^6^ RBMX-knockout or control cells suspended in 50% Matrigel. There were four animals in each group. Tumor growth was assessed every three days using caliper measurements, with the tumor volume calculated using the following formula: Volume = 0.5 × length × width^2^. Once the tumor volume reached 1000 mm^3^ [29], the mice were euthanized with carbon dioxide and tumors were collected for further analysis.

### 2.11. Cell Coculture

Spleens were harvested from 6-week-old mice (BALB/c mice were used in the K7M2 cell line, and C3H mice were used in LM8) and mechanically dissociated through a 70 μm nylon mesh. After depleting erythrocytes with ACK lysing buffer (Gibco, New York, NY, USA), mouse CD8^+^T cells were isolated by negative selection via the EasySep Isolation System (StemCell Technologies, Vancouver, BC, Canada).

CD8^+^T cells were cultured in RPMI-1640 medium with 10% heat-inactivated FBS, 1% antibiotic–antimycotic solution, 50 μM β-mercaptoethanol, and 20 U/mL recombinant murine IL-2 (PeproTech, Cranbury, NJ, USA). Cells were stimulated for 48 h using plates coated with anti-CD3 (5 μg/mL, BD, Franklin Lakes, NJ, USA) and soluble anti-CD28 (2 μg/mL, BD) antibodies. Then, the activated CD8^+^T cells were collected and cocultured with tumor cells in 96-well plates (1:10) for 72 h. Cytokine production was evaluated by restimulating activated CD8^+^T cells with 10 μg/mL brefeldin A (Thermo Fisher) for 4 h at 37 °C. Cells were harvested and cytokines were detected via intracellular staining with anti-IFN-γ-PE and anti-GZMB-APC antibodies (Biolegend, San Diego, CA, USA) and analyzed using a flow cytometer.

### 2.12. Tumor Cell Dissociation and Staining

Subcutaneous tumor tissues were harvested from mice at the experimental endpoint and immediately placed in cold PBS. The tissues were cut into 1–2 mm^3^ pieces using sterile scissors and digested in RPMI 1640 medium with 1 mg/mL collagenase D (Roche, Basel, Switzerland) and 20 μg/mL DNase I (Sigma, Darmstadt, Germany) at 37 °C for 30–45 min with gentle agitation. The digested tissue was then passed through a 70 μm cell strainer to obtain a single-cell suspension. Red blood cells were lysed with ACK lysis buffer (Gibco) for 2 min at room temperature and then washed with PBS containing 2% FBS. For flow cytometry analysis, cells were resuspended in PBS and stained in the dark at 4 °C for 30 min using fluorochrome-conjugated antibodies: CD45-BV510, CD3ε-PerCP-Cy5-5, CD8α-FITC, and CD4-APC-R700 (all from Biolegend).

### 2.13. Statistical Analysis

Statistical significance was determined using a two-tailed hypothesis test with an α threshold of 0.05. The normality of data distribution was assessed by the Shapiro–Wilk test (for sample size < 50) or Kolmogorov–Smirnov test (for sample size ≥ 50). Parametric tests (e.g., Student’s *t*-test) were used for normally distributed data, whereas non-parametric tests (e.g., Mann–Whitney U test) were applied otherwise. For experiments involving multiple comparisons, the false discovery rate (FDR) was controlled using the Benjamini–Hochberg method. Animal experiments included four mice per group, and cell experiments were performed with three replicates per group. Overall survival was analyzed by the Kaplan–Meier method, with statistical significance assessed by the log-rank test. All analyses were conducted using R software (v4.3.0) and GraphPad Prism (v10).

## 3. Results

### 3.1. RBMX Could Serve as an Indicator of Poor Prognosis in Osteosarcoma Patients

**Figure 1:**
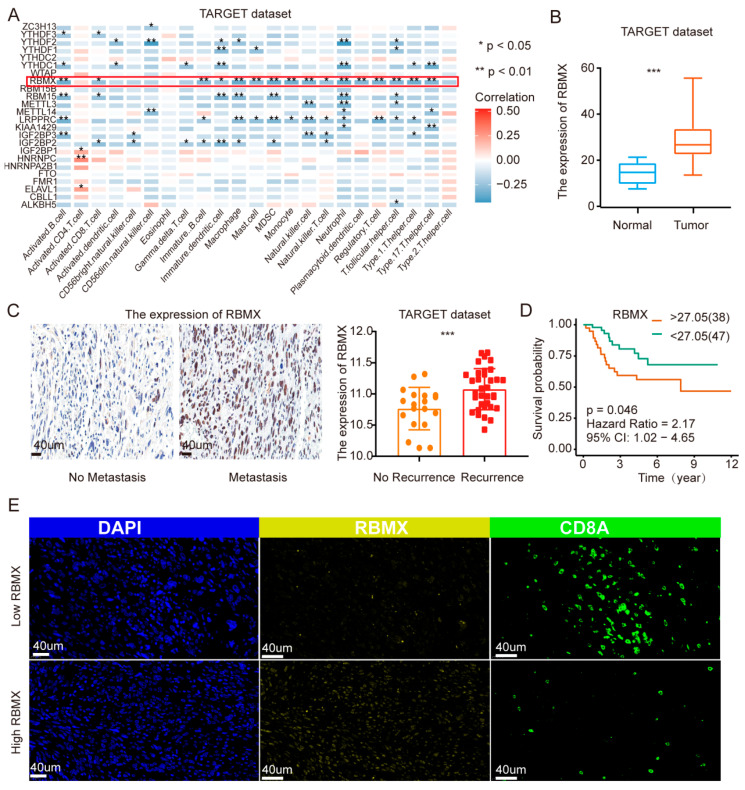
RBMX is a factor for poor prognosis in human osteosarcoma. (**A**) The TARGET database demonstrated that the expression of RBMX was significantly negatively correlated with the infiltration levels of most cells in the human osteosarcoma microenvironment. (**B**) Transcriptome sequencing analysis revealed that RBMX was highly expressed in human osteosarcoma tissues. (**C**) Protein levels and RNA levels revealed that high expression of RBMX might promote the metastasis of human osteosarcoma cells. (**D**) The survival curve reveals that a high expression of RBMX correlates with a poor prognosis for human osteosarcoma. (**E**) Multiplex immunofluorescence revealed that in human osteosarcoma tissues with a high expression of RBMX, the infiltration level of CD8^+^T cells decreased significantly. * *p* < 0.05, ** *p* < 0.01, *** *p* < 0.001.

Through ssGSEA enrichment analysis and correlation analysis, we found that, among all the m6A modified regulatory factors, the expression of RBMX was negatively correlated with most immune cells in the osteosarcoma TME, which suggested that RBMX may be a negative regulatory factor in the osteosarcoma microenvironment (Figure 1A). Transcriptome sequencing analysis revealed that RBMX was highly expressed in osteosarcoma tissues (Figure 1B). The immunohistochemical staining showed that RBMX expression was elevated in metastatic and recurrent tumor tissues compared to non-metastatic and non-recurrent cases (Figure 1C). In addition, osteosarcoma patients with a high expression of RBMX had a poorer prognosis (Figure 1D). Furthermore, through multiplex immunofluorescence staining, we found that, in the tissues with a high expression of RBMX, the infiltration level of CD8^+^T cells decreased significantly (Figure 1E). The above results indicated that RBMX might play an important role in osteosarcoma progression, be an adverse prognostic factor for osteosarcoma, and be involved in inhibiting the osteosarcoma microenvironment.

### 3.2. Downregulation of RBMX Inhibits the Proliferation of Osteosarcoma Cells

**Figure 2:**
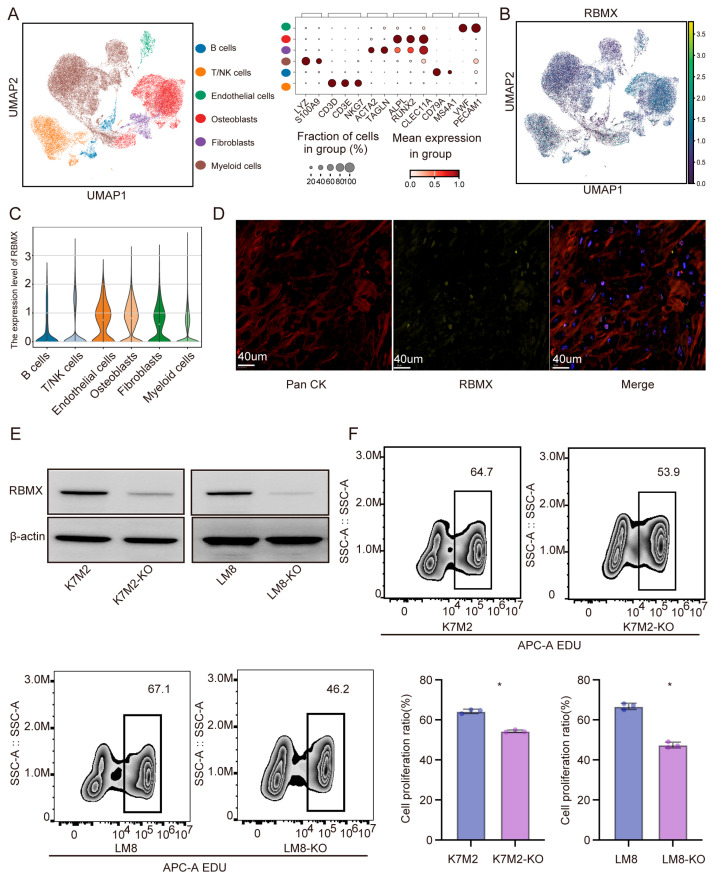
Knockout of RBMX expression in osteosarcoma cells significantly inhibited the growth of tumor cells. (**A**) The UMAP diagram shows the cell clustering of human osteosarcoma. (**B**,**C**) Analysis of RBMX gene expression in human osteosarcoma tissues. (**D**) PAN-CK and RBMX expression in human osteosarcoma tissues were analyzed using multiplex immunofluorescence. (**E**) The expression of RBMX in the K7M8 and LM8 cell lines (mouse osteosarcoma cell line) was analyzed using Western blot. (**F**) The incorporation rate of EDU was determined via flow cytometry. Student *t*-tests were used for statistical analysis of the differences in each group. * *p* < 0.05.

To understand the effects of RBMX on the progression of osteosarcoma, we analyzed the single-cell transcriptome of human osteosarcoma tissue (GSE162454) (Figure 2A,B). As shown in Figure 2C,D, RBMX is widely expressed in epithelial cells. Following infection with RBMX-knockout lentivirus, the RBMX expression in mouse osteosarcoma cells was significantly decreased compared with the empty group (Figure 2E). The incorporation rate of EdU was significantly decreased in the RBMX knockout cells, as compared to the empty control, suggesting that downregulation of RBMX inhibited osteosarcoma cell proliferation (Figure 2F).

### 3.3. Knockout of RBMX Significantly Activates the Immune Microenvironment of Osteosarcoma

**Figure 3:**
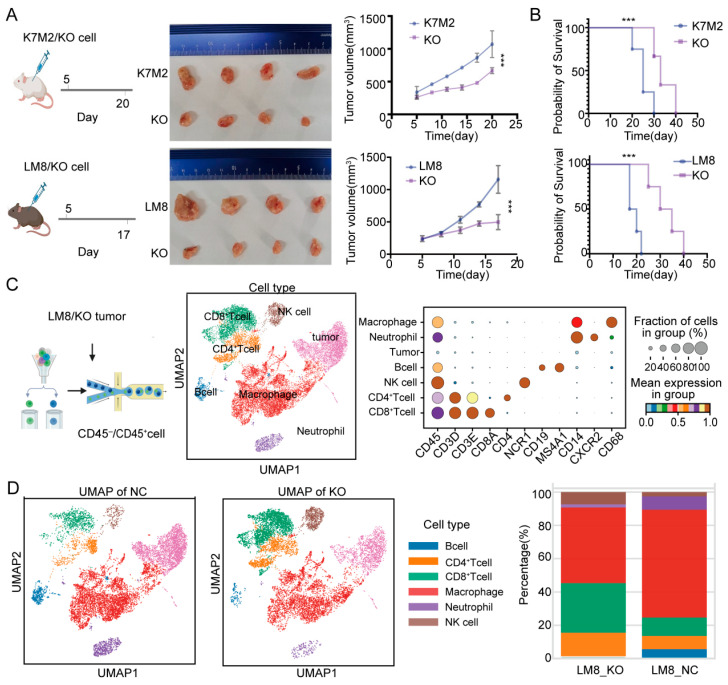
Knockout of RBMX remodeled the mouse osteosarcoma microenvironment. (**A**) Subcutaneous implantation of both K7M2 and LM8 mouse osteosarcoma models revealed that RBMX knockout significantly inhibited tumor growth (four mice were used in each group). Compared to their respective control groups (1070.25 ± 201.07 mm^3^ for K7M2 and 1158.00 ± 190.24 mm^3^ for LM8), the average tumor volume in the RBMX-knockout group was markedly reduced to 666.75 ± 41.98 mm^3^ and 496.75 ± 105.00 mm^3^, respectively. (**B**) In the K7M2 subcutaneous tumor model, RBMX knockout significantly prolonged survival. The median survival time was 25 days in the control group compared to 37.5 days in the RBMX-knockout group. The hazard ratio for death was 0.25 (95% confidence interval: 0.07 to 0.90). Similarly, in the LM8 model, RBMX knockout also significantly improved survival outcomes. The median survival time was 18.5 days in the control group compared to 32.5 days in the RBMX-knockout group. The hazard ratio for death was 0.19 (95% confidence interval: 0.04 to 0.88). (**C**) Single-cell transcriptome sequencing of subcutaneous implantable tumors in mice. (**D**) UMAP plots and overlay plots reveal the differences in the proportion of immune cells in the TME between LM8 and LM8_KO subcutaneous implantable tumors. *** *p* < 0.001.

**Figure 4:**
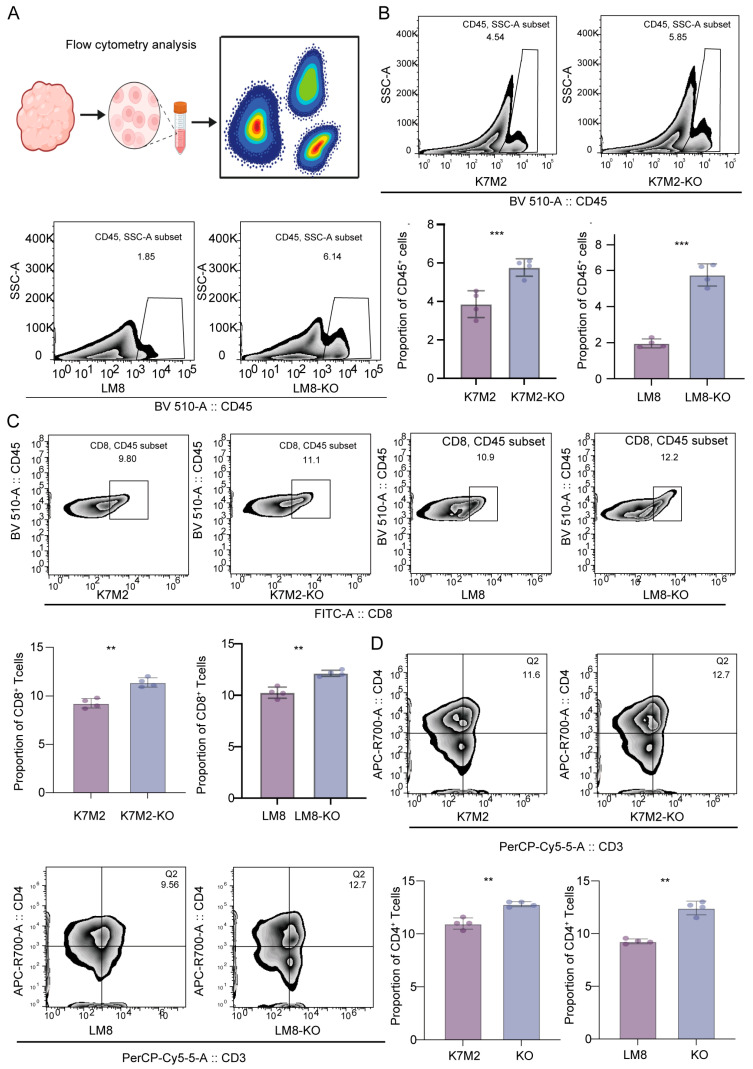
RBMX knockout enhances the infiltration of immune cells in the mouse osteosarcoma TME. (**A**) The subcutaneous tumor tissues of mice were dissected for flow cytometry analysis. (**B**) The infiltration level of CD45^+^ cells in mouse tumor tissues was analyzed. (**C**) The infiltration level of CD8^+^T cells in tumor tissues. (**D**) The infiltration level of CD4^+^T cells in tumor tissues. ** *p* < 0.01, *** *p* < 0.001.

By constructing a mouse subcutaneous tumor model, we determined that knockout of RBMX significantly inhibited the growth of subcutaneous implant tumors, and the mice had a longer survival time (Figure 3A,B). To further understand the effects of RBMX knockout on the osteosarcoma immune microenvironment, we separated the tumor tissue and isolated single cells for transcriptome sequencing. As shown in Figure 3C,D, RBMX knockout led to an increase in T and NK cells, along with a decrease in macrophages. This result suggested that RBMX may be an immunosuppressive factor for osteosarcoma, since single-cell transcriptome sequencing was only performed in models constructed from the LM8 cell line. For this purpose, flow cytometry analysis was conducted on subcutaneous tumor tissues constructed from two cell lines (LM8 and K7M2) in this study (Figure 4A). The results showed that in the LM8 and K7M2 cell lines, knockout of RBMX significantly increased the proportion of CD45^+^ cells in subcutaneous tumor tissues (Figure 4B). Further analysis revealed that knockout of RBMX significantly increased the proportion of CD8^+^T cells in immune cells of subcutaneous tumor tissues (Figure 4C). Similarly, knockout of RBMX significantly increased the proportion of CD4^+^T cells in immune cells of subcutaneous tumor tissues (Figure 4D).

### 3.4. CD8^+^T Cells Might Mediate the Suppressive Capacity of RBMX Knockout in the Osteosarcoma Microenvironment

**Figure 5:**
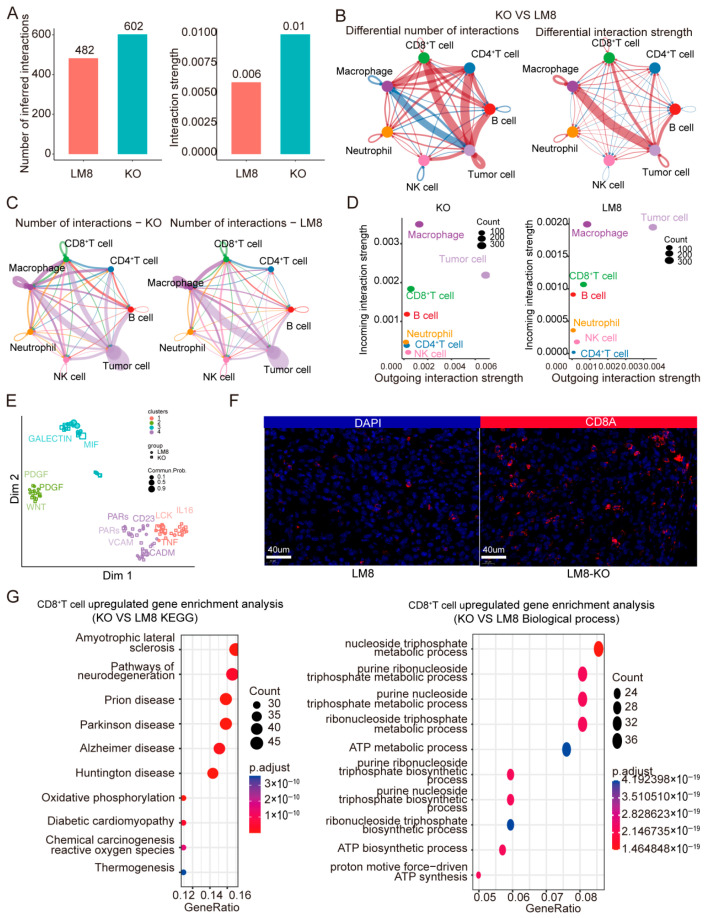
CD8^+^T cells might be an important cell subset regulated by RBMX. (**A**) The number and intensity of cell communications in the mouse TME were analyzed using CellChat. (**B**) The string plot shows the differences in the number and intensity of communications among various cell subsets. Red indicates that the KO group is high, and blue indicates that the control group is high. (**C**) The string plot shows the number of communications between each cell subpopulation in each group. (**D**) This figure shows the changes in the receptors emitted and received by different cell groups in different groups. (**E**) This figure shows the potential functions of different groups based on the results of cell communications. (**F**) The infiltration of CD8^+^T cells in the tumor was analyzed using multiplex immunofluorescence. (**G**) Functional enrichment analysis using KEGG and GO is indicated.

To explore which types of cells might be regulated by RBMX, CellChat analysis was conducted. The RBMX knockout group exhibited a greater number and intensity of ligand pairs compared to the control group (Figure 5A). Further analysis indicated that in the knockout group, tumor cells exhibited a higher quantity and intensity of communication with T cells and B cells than in the control group, whereas their interaction with macrophages was reduced (Figure 5B,C). Further analysis indicated that the signal intensity from the tumor cells and the signals exchanged by CD8^+^T cells were higher in the knockout group compared to the control (Figure 5D). Functional analysis showed that the knockout group was widely enriched in immune-related modules (Figure 5E). Based on the above results, we speculate that CD8^+^T cells may mainly mediate the roles of RBMX, and this result was further confirmed through immunization (Figure 5F). The analysis revealed that CD8^+^T cells in the knockout group exhibited significant enrichment in energy metabolism-related pathways compared to the control group (Figure 5G).

### 3.5. RBMX Knockout Promoted CD8^+^T Cell Infiltration in the Osteosarcoma Microenvironment

**Figure 6:**
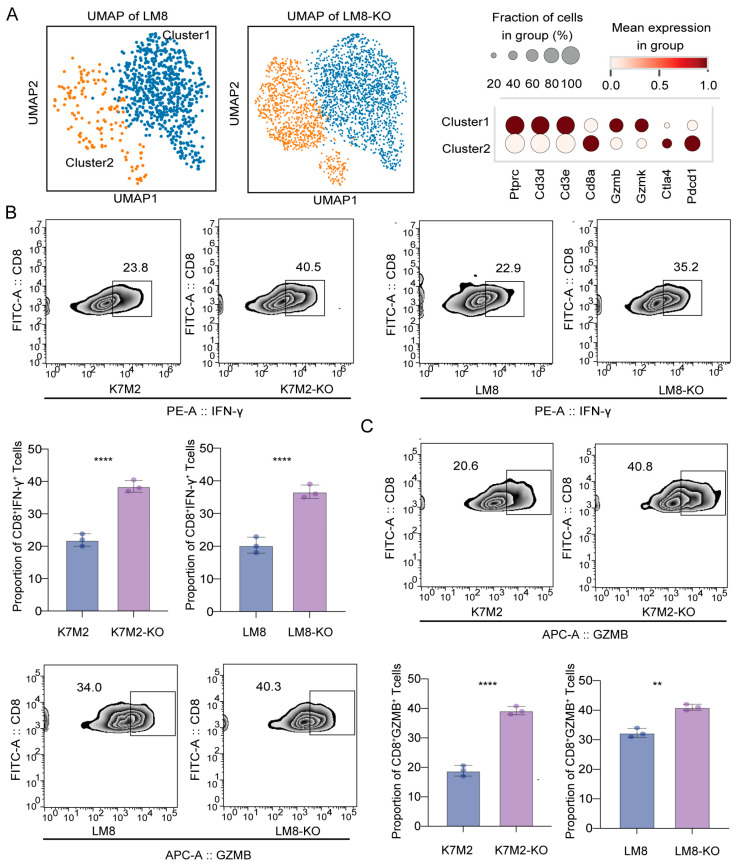
Knockout of RBMX significantly enhances the cytotoxicity of CD8^+^T cells. (**A**) The UMAP diagram shows the distribution of CD8^+^T cell subsets in different groups. (**B**) The capacity of CD8^+^T cells to express IFN-γ in the osteosarcoma microenvironment was analyzed. (**C**) The capacity of CD8^+^T cells to express Granzyme B (GZMB) in the osteosarcoma microenvironment was analyzed. ** *p* < 0.01, **** *p* < 0.0001.

**Figure 7:**
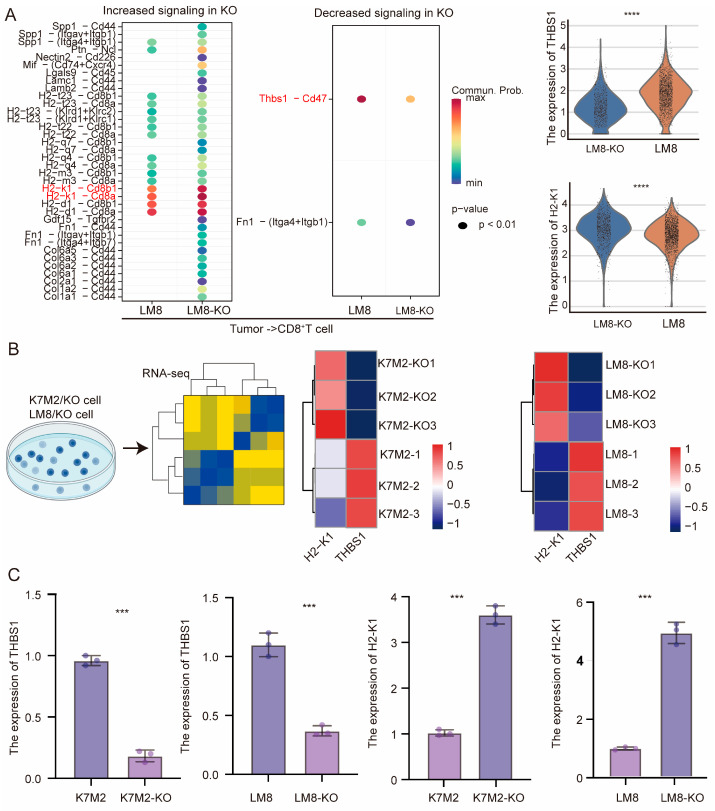
RBMX knockout could promote CD8^+^T cell infiltration in the mouse osteosarcoma microenvironment via H2-K1 and THBS1. (**A**) Enhanced and weakened signaling pathways in the TME after RBMX knockout were analyzed using cell communication analysis. (**B**) RNA transcriptome sequencing analysis. (**C**) mRNA expression of RBMX, H2-K1, and THBS1 in the osteosarcoma microenvironment was detected. *** *p* < 0.001, **** *p* < 0.0001.

The impact of RBMX knockout on CD8^+^T cells within the osteosarcoma microenvironment is not yet understood. Single-cell transcriptome sequencing revealed that the knockout group exhibited increased numbers of both exhausted (CTLA4^hig^PDCD1^hig^CD8^+^ T cells) and cytotoxic CD8^+^T cells (GZMB^high^GZMK^high^CD8^+^ T cells) compared to the control group (Figure 6A). After stimulation in vitro, splenic CD8^+^T cells were directly cocultured with tumor cells from different groups. The proportion of the cytotoxic molecules IFN-γ and Granzyme B in CD8^+^T cells was higher in the knockout group than in the control, suggesting that RBMX knockout in tumor cells may influence CD8^+^T cell activity via cellular interactions (Figure 6B,C). Furthermore, we utilized CellChat to investigate the impact of RBMX knockout on CD8^+^T cell activity in the TME. As shown in Figure 7A, there was a significant enhancement in the H2-K1 signaling pathway and a notable weakening of the THBS-1 signaling pathway between tumor cells and CD8^+^T cells following RBMX knockout; the expression of H2-K1 in tumor cells significantly increased, while the THBS-1 expression was decreased (Figure 7A). Both transcriptome sequencing and mRNA expression via PCR also showed a similar trend (Figure 7B,C). Collectively, these results indicate that RBMX knockout in tumor cells boosts CD8^+^T cell activity by promoting H2-K1 and inhibiting THBS-1 expression.

## 4. Discussion

Osteosarcoma is a common malignant tumor in bone, which is associated with a poor prognosis [1]. Although innovations in comprehensive treatments such as immunotherapy, targeted therapy, chemotherapy, and surgery have prolonged the survival time, the prognosis remains unsatisfactory [3]. Therefore, exploring new therapeutic targets and enhancing therapeutic efficacy to improve the prognosis of patients are important tasks in current research.

The TME is a critical determinant of osteosarcoma patient survival [30]. Studies indicate that osteosarcoma cells promote progression by increasing M2 macrophage polarization within the TME [31]. Osteosarcoma patients with a high infiltration of effector T cells show a higher response to treatment and a more favorable prognosis [32]. Relevant studies have found that the increase in the expression of tumor-infiltrating T cells and PD-L1 in metastatic osteosarcoma is significantly higher than that in the primary lesion, suggesting that patients with metastatic osteosarcoma may benefit from T cell-based immunotherapy [33]. Therefore, exploring ways to improve the microenvironment of osteosarcoma is urgent.

N6-Methyladenosine modification, representing the most abundant epitranscriptomic mark in mammalian cells, serves as a pivotal regulator of immunological processes in cancer, including immune effector functions, chemokine/cytokine cascades, and MHC-mediated antigen display [34]. Recent studies have shown that N6-Methyladenosine modification writers, erasers, and readers dynamically affect the TME [35]. The N6-Methyladenosine modification regulatory factor influences osteosarcoma progression by modulating the TME [36]. Through investigating N6-Methyladenosine modification regulatory factors in the osteosarcoma TME, this study found that the expression of RBMX was significantly negatively correlated with the infiltration level of CD8^+^T cells. This result indicates the potential value of RBMX. Also known as HNRNPG heterogeneous ribonucleoprotein, RBMX is an RNA-binding protein and a member of the endonuclear heterogeneous ribonucleoprotein family [37]. Relevant research reports indicate that RBMX is involved in regulating selective splicing, chromatid cohesion and genomic instability [38]. Relevant studies have found that RBMX may be a carcinogenic factor and correlates with poor prognosis [39]. RBMX regulates the mTOR signaling pathway by stabilizing CPT1A and participates in lipid metabolism and proliferation in chronic lymphocytic leukemia, promoting the progression of the disease [40]. However, research on RBMX in osteosarcoma is still unclear, and there are few related reports on the immune microenvironment. Prior research has primarily concentrated on RBMX’s involvement in RNA splicing and DNA damage repair [41]. This study systematically revealed, for the first time, the key role of RBMX in the regulation of the immune microenvironment of osteosarcoma. The analysis of clinical samples and experimental data revealed that RBMX is significantly overexpressed in osteosarcoma tissues and is closely associated with poor patient prognosis. Notably, these findings indicate that elevated RBMX expression is closely linked to an immunosuppressive microenvironment, characterized by decreased CD8^+^T cell infiltration and increased tumor-associated macrophages. This result reveals that RBMX is involved in the regulation of the tumor microenvironment of osteosarcoma and may be an important target for adjusting the state of the tumor microenvironment.

However, the mechanism by which RBMX specifically regulates the TME state of osteosarcoma remained unclear. To explore this mechanism, this study used single-cell transcriptome sequencing combined with multi-omics analysis to determine that knockout of RBMX expression significantly upregulated the expression of H2-K1 and downregulated the expression of THBS1 in osteosarcoma cells. H2-K1 is associated with the major histocompatibility complex Class I (MHC-I) [42]. The induced expression of H2-K1 significantly enhances tumor immunogenicity and sensitivity to immune checkpoint inhibitors [43]. H2-K1 promotes NK cell maturation by interacting with ligands on NK cells [44]. Relevant studies have also found that the expression of H2-K1 in mouse tumor cells can induce CD8^+^ T cell responses in mice and kill tumor cells [42]. The above results suggest that the knockout of RBMX in osteosarcoma can promote the expression of H2-K1 and may activate the activity of CD8^+^T cells in osteosarcoma tissue through ligand action, thereby mediating the tumor-killing effect. THBS1 is a family composed of five glycoproteins that interact with growth factors and cytokines for intercellular communication [45]. THBS1 knockout can restore genes related to antigen expression, promote the infiltration of pro-inflammatory TAM and CD8^+^T cells in tumors, and alleviate TAM-mediated T cell inhibition [46]. Relevant studies have found that THBS1 in tumors can promote tumor metastasis by inducing cytotoxic T cell depletion [47]. Tumor cells inhibit the antitumor activity of CD8^+^T cells by coordinating the MGAT1-CD73-VAMP3-adenosine axis through THBS1 [48]. The above results suggest that, in osteosarcoma, RBMX knockout downregulates the expression of THBS1, which may further inhibit the activity of CD8^+^T cells through cell communication.

However, this study also has some limitations. Firstly, in this study on the impact of RBMX expression on prognosis in osteosarcoma, due to the lack of corresponding clinical information, it is impossible to accurately reflect whether RBMX is an independent prognostic factor for osteosarcoma. This may require further collection of more clinical samples for exploration in the future. Secondly, this study found that RBMX is highly expressed in osteosarcoma and may promote tumor progression by inhibiting the activity of CD8^+^T cells. This study lacks rescue experiments and requires further exploration. In addition, no inhibitors or drugs that can regulate RBMX expression have been discovered. Targeted therapy for RBMX is currently in the preclinical stage and requires safety evaluation. Further screening of drugs that can regulate RBMX expression may be needed in the future to provide possibilities for clinical treatment. Thirdly, this study did not include a fixable viability dye in the flow cytometry staining panel. Although cell viability was high at the time of staining, as assessed by trypan blue exclusion, we cannot fully rule out the potential contribution of dead cells to nonspecific antibody binding. Future studies will incorporate viability staining to ensure the highest purity of analyzed populations.

## 5. Conclusions

The results of this study revealed that RBMX is an important regulatory factor of the osteosarcoma microenvironment. Targeting RBMX could allow for alteration of the osteosarcoma microenvironment, thus inhibiting its progression, and offer novel targets and strategies for precise immunotherapy.

## Data Availability

The transcriptome sequencing data and bulk data of human osteosarcoma in this study were derived from the GEO (GSE162454) and TARGET databases (https://ocg.cancer.gov/programs/target, accessed on 15 December 2023). The raw data of the bulk transcriptome sequencing of mouse cell lines and single-cell transcriptome sequencing of mouse subcutaneous tumor tissues in this study will be made available by the authors on request.
